# Reading networks in children with dyslexia compared to children with ocular motility disturbances revealed by fMRI

**DOI:** 10.3389/fnhum.2014.00936

**Published:** 2014-11-19

**Authors:** Ibone Saralegui, José M. Ontañón, Begoña Fernandez-Ruanova, Begonya Garcia-Zapirain, Alejandro Basterra, Ernesto J. Sanz-Arigita

**Affiliations:** ^1^Department of Neuroradiology, Osatek, Galdakao-Usansolo HospitalGaldakao, Spain; ^2^Research Department, OsatekBilbao, Spain; ^3^DeustoTECH Life (eVIDA), University of DeustoBilbao, Spain; ^4^CITA-Alzheimer FoundationDonostia, Spain; ^5^Radiology and Image Analysis Centre, VU Medical CentreAmsterdam, Netherlands

**Keywords:** developmental dyslexia, fMRI, ocular motility disorders, paradigm, pseudoword

## Abstract

**Key Points**

- Dyslexia is a neurological disorder with a genetic origin, but the underlying biological and cognitive causes are still being investigated.
- This study compares the brain activation pattern while reading in Spanish, a semitransparent language, in three groups of children: typically developing readers, dyslexic readers and readers with functional monocular vision.
- Based on our results Dyslexia would be a neurological disorder not related to vision impairments and would require a multidisciplinary treatment based on improving phonological awareness and language development.

Developmental dyslexia is a neurological disorder the underlying biological and cognitive causes of which are still being investigated, a key point, because the findings will determine the best therapeutic approach to use. Using functional magnetic resonance imaging, we studied the brain activation pattern while reading in the language-related cortical areas from the two reading routes, phonological and orthographic, and the strength of their association with reading scores in 66 Spanish-speaking children aged 9–12 years divided into three groups: typically developing readers (controls), dyslexic readers and readers with monocular vision due to ocular motility disorders but with normal reading development, to assess whether (or not) the neuronal network for reading in children with dyslexia has similarities with that in children with impaired binocular vision due to ocular motility disorders. We found that Spanish-speaking children with dyslexia have a brain circuit for reading that differs from that in children with monocular vision. Individuals with dyslexia tend to hypoactivate some of the language-related areas in the left hemisphere engaged by the phonological route, especially the visual word form area and left Wernicke's area, and try to compensate this deficit by activating language-related areas related to the orthographic route, such as the anterior part of the visual word form area and the posterior part of both middle temporal gyri. That is, they seem to compensate for impairment in the phonological route through orthographic routes of both hemispheres. Our results suggest that ocular motility disturbances do not play a causal role in dyslexia. Dyslexia seems to be a neurological disorder that is unrelated to vision impairments and requires early recognition and multidisciplinary treatment, based on improving phonological awareness and language development, to achieve the best possible outcome.

## Introduction

Language, unlike reading, is predefined in our genome. Indeed, language acquisition is natural and inherent to the human species. In contrast, writing and also reading, viewed from an evolutionary perspective, are very recent inventions (Artigas-Pallarés, 2011).

The human brain is not intrinsically literary, therefore to incorporate these skills it is necessary to use brain structures not designed for such functions by natural selection. Dehaene proposed the theory of “neuronal recycling” to describe such adaptations in the function of an organ. This is the case of the fusiform gyrus, a region used in primates and other species to display visual forms (e.g., predators, prey or potential mates), that has been adapted in humans to visualize the shapes of the letters of the alphabet (Paulesu et al., 2001; Dehaene and Cohen, 2007; Dehaene et al., 2010). On the other hand, recent studies suggest that nearly 20% of the population has some degree of learning disability, in many cases attributable to reading difficulties, reading being a complex cognitive process that is required for complicated and sophisticated learning (National Center for Learning Disabilities, 2010)^1^.

Dyslexia is the most prevalent learning disability (80%) (Handler et al., 2011). The term dyslexia is derived from the Greek. δυσλεξíα (dyslexia), formed by the prefix δυς (dys- = wrong, with difficulty), λέξις (lexis = word) and the suffix –íα (-ia = quality). It is defined as “a disorder manifested by difficulty learning to read, despite conventional instruction, adequate intelligence and sociocultural opportunity” (World Health Organization, ICD-10). This means that 700 million people worldwide have features of dyslexia and are at risk of life-long illiteracy and social exclusion if their dyslexia is not properly addressed.

It is now well established that dyslexia is a neurological disorder with a genetic origin, which is currently being investigated. Beyond this consensus, the underlying biological and cognitive causes of the reading retardation are still debated (Willcutt and Pennington, 2000; Ramus et al., 2003; Démonet et al., 2004; Serrano and Delfior, 2004; Ramus, 2006).

Although there is now a strong consensus among researchers in the field that the central difficulty in dyslexia reflects a deficit within the language system (Phonological theory, Galaburda et al., 1985; Shaywitz et al., 1998; Snowling and Hulme, 2011: Paulesu et al., 2001), other theoretical models remain compelling, such as the Auditory temporal processing deficit theory (Tallal et al., 1996), the Cerebellar theory (Nicolson et al., 2001), and more recently the Visual attention span deficit theory (Roach and Hogben, 2004; Facoetti et al., 2006; Bosse et al., 2007; Lobier et al., 2012), and the Magnocellular visual deficit theory of dyslexia (Livingstone et al., 1991; Stein and Walsh, 1997; Vidyasagar and Pammer, 2009). The last of these postulates that the magnocellular pathway is selectively disrupted in certain dyslexic individuals, and that this leads to deficiencies in visual processing. The visual theory does not exclude a phonological deficit, but emphasizes an additional visual contribution to reading problems, at least in some dyslexic individuals.

The diverse theories proposed and the different patterns of performance observed have led several researchers to consider developmental dyslexia to be a heterogeneous impairment resulting from independent cognitive disorders, with a majority subtype suffering from a phonological deficit, and a minority characterized by a visual deficit (Vellutino et al., 2004; Bosse et al., 2007).

Many authors in the optometric literature, as opposed to the ophthalmologic literature defend the view that children with reading disorders have an increased incidence of vision abnormalities and proclaim the usefulness of vision therapy for reading and learning disabilities (Irlen, 1983; Skeffington, 1988; Solan et al., 1998), despite it not having been proven that there is a significant difference in reading ability between readers with normal and abnormal binocular function (Grisham et al., 1993). Other studies have also been unable to find an increase in the incidence of binocular disorders in children with reading difficulties or an association between motility disorders and reading ability (Hall and Wick, 1991).

Abnormal eye tracking has also been mistakenly implicated as having a causative role in reading problems. Indeed, individuals with an almost complete inability to move their eyes show normal reading ability, however (Hodgetts et al., 1998). From an ophthalmological point of view, individuals with dyslexia show many of the same types of eye movements as a beginning reader, but as dyslexics show normal sequential saccade tracking in other areas of oculomotor functioning, it is believed that the abnormalities seen in individuals with dyslexia during reading are a result, and not the cause, of their reading disability (Rayner et al., 1996; Hoyt, 1999; Olitsky and Nelson, 2003). That is, decoding and comprehension difficulties, rather than a primary abnormality of the oculomotor control systems, are responsible for slow reading, increased duration of fixations, and increased backward saccades (Hoyt, 1999). Recent studies based on fMRI results support the hypothesis that visual magnocellular dysfunction would be the consequence and not the cause of reading disabilities (Olulade et al., 2013).

The aim of the present research was to analyse the neural network while reading in a group of children with dyslexia and compare it with the network obtained in two other groups, one of children with typical development, and children with monocular vision secondary to ocular motility disorders, who have impaired stereopsis and saccadic eye movements in binocular vision. A main objective was to assess whether dyslexic readers share neuronal patterns with children with ocular motility disorders; if, in contrast, there are differences in their reading networks, ocular motility disorders should not be considered a direct cause of dyslexia.

For this purpose we have conducted a comprehensive fMRI study including three different cognitive paradigms in order to explore the two main routes of reading, phonological and orthographic (Colheart et al., 2001). Specifically, we included two paradigms of lexical decision to elicit the activation of the phonological network, and we further tested the linguistic abilities with the inclusion of a specific paradigm for semantic categorization to activate the orthographic route, in which the subject has to create a conceptual representation of two cue words, find their relationship and compare it with a target word in order to determine if it belongs to the same category.

High resolution functional magnetic resonance imaging of the brain activity patterns elicited by this set of reading-based paradigms might help to distinguish the underlying mechanisms of dyslexia and its relation with visual impairment, with beneficial consequences for the diagnosis and treatment of deficits of the reading system and reading retardation in particular.

Additionally, we wanted to evaluate which of the three paradigms was the most reliable for studying dyslexia, analysing the significant differences in cortical activations between children with dyslexia compared to children with typical reading development, and correlating them with the scores of the standardized clinical assessments for the evaluation of reading processes, until now the gold standard for the diagnosis of dyslexia.

## Materials and methods

### Ethics statement

This research has been performed under the Code of Ethics of the World Medical Association (Declaration of Helsinki) and the standards established and approved by the Clinical Research Ethics Committee at Galdakao Hospital, which included an informed consent form being signed by the parents or guardians of each participant before inclusion. In addition, all participants were informed about the study purposes and protocols.

### Participants

Sixty-six children between 9 and 12 years of age were recruited from the Departments of Pediatric Ophthalmology and Neurology, at Cruces University Hospital, and from schools in the same area (Bilbao, Spain) in the case of controls, after their parents gave written informed consent.

Three age-matched reading groups were prospectively selected according to the following selection criteria:

**Inclusion criteria:** Children were to be between 9 and 12 years of age and right-handed (left-handed participants were not included in the study to avoid laterality effects); as well as have Spanish was their mother tongue and an IQ within the normal range, considered as to be a Wechsler Intelligence Scale for Children—Fourth Edition (WISC-IV; Wechsler, 2005) Full Scale IQ > 75. In addition, for the dyslexic group, children were required to have a diagnosis of dyslexia without having received treatment or psycho-pedagogical support for literacy; and all children assigned to the monocular vision group (on the basis of opthalmological assessments, described below) were typical readers.

**Exclusion criteria:** We excluded those with previous history of neurological disease or severe head trauma, impaired sensory-motor coordination, psychiatric illness, chronic drug treatments, social deprivation, inadequate schooling or intolerance to MRI scanning (claustrophobia, or a lack of cooperation, among other factors); and candidates for the dyslexic and the control groups were excluded if they had any abnormalities in vision, except for a refractive error corrected with normal visual acuity, or had any motility abnormalities on clinical examination.

### Ophthalmological measures

A pediatric ophthalmologist examined the children to select the group with monocular vision secondary to ocular motility disorders and to detect any kind of ophthalmological problems in candidates from the other two groups. The examination included testing of far and near visual acuity (both spontaneous and corrected); far binocular vision (using the Worth and vectographic tests) and near binocular vision (with the TNO test); ocular motility (with alternate cover and cover/uncover tests), and visual acuity (with cycloplegic refraction), as well as slit lamp and eye fundus examinations.

The group of readers with monocular vision secondary to ocular motility disorders (MVR) included children with strabismus (*n* = 13), monocular microphthalmia (1 girl), nystagmus (*n* = 2) and paralysis of the extraocular muscles (Fell's syndrome) (1 girl). All these children had a functional monocular vision due to a suppression phenomenon of the non-dominant eye and suffered from an impairment of the binocular coordination of saccades (Kapoula et al., 1997; Bucci et al., 2002).

In the case of the other two groups, children were excluded from the study if they had any notable ophthalmological problems such as visual acuity less than 20/20, far binocular vision problems, TNO less than 60″, anomalies in the slit lamp or fundus examinations or refractive errors (hypermetropia > 3.5D, any myopia magna, or astigmatism > 1.5D).

### Behavioral measures

A pediatric neuropsychologist evaluated all the children using three types of tests:

Initially, as mentioned above, intelligence was measured with the WISC-IV. All children had IQ scores of over 75, which ruled out intellectual disabilities.

Secondly, reading-related skills were evaluated with a series of standardized reading tests, including a battery for the evaluation of reading processes (PROLEC-R for 8- to 11-year-olds or PROLEC-ES for those ≥12 years) (Cuetos et al., 2007), and in some borderline cases, an evaluation of reading comprehension (ECLE-2) (De la Cruz, 1997) for word reading fluency in terms of accuracy and speed. Children were classified as dyslexic readers based on a standardized score 2 standard deviations below (<−2 SD) the expected means in the evaluations of reading processes and comprehension. Typical readers (the controls and those with monocular vision) scored above the mean on all tests. As noted above, any children who had received prior specific remedial treatment for dyslexia were not included.

Thirdly, behavior was evaluated using the Behavior Assessment System for Children (BASC) (González et al., 2004) to identify and exclude children with any psychological, profound sensory or neurological impairment, as well as impairment in family or academic functioning. In addition, to determine the lateral dominance in cases of doubt the Harris Hemispheric Dominance test (Harris, 2001) was used. Left-handed participants were not included in the study.

Based on the ophthalmologist's examination and readers' standardized test performance, children were assigned to one of the three reading groups: Typically Developing Readers (TDRs, the controls), Dyslexic Readers (DXRs) or readers with Monocular Vision secondary to ocular motility disorders (MVRs). Groups were matched for age.

In total, 19 children (11 boys) were categorized into the DXR group, 17 (10 boys) into the MVR group and 19 (11 boys) into the TDR group. Five children with ADHD (Attention Deficit Hyperactivity Disorder) were included in our study, 2 in the DXR group, 1 in the MVR group and 2 in the TDR group. From the initial number of participants recruited, 11 children were excluded: 7 children with reading problems but with scores 2 standard deviations above the expected means in the evaluations of reading processes and comprehension, 1 of them with an IQ of 69; 1 left-handed child; and another 3 due to unrecoverable data from the functional MR scan.

Table 1 lists descriptive statistics for the three groups, including performance scores on behavioral measures assessing phoneme awareness. Consistent with the definition of the groups, performance on word and pseudoword reading was significantly poorer in the DXR group than in the other two groups (TDR and MVR).

**Table 1:** **Subject characteristics**.

	**TDR**	**DXR**	**MVR**	P^*^
Sample size	19	19	17	
Age (years) ^**^	10.0 (0.9)	10.6 (1.0)	10.5 (0.9)	0.151
Gender (Female/Male)	8/11	8/11	7/10	0.998
Attention deficit hyperactivity disorder	2	2	1	0.858
Corrected visual acuity	1.00	1.00	1.00	
IQ^**^				
Full scale	108.3 (12.0)	94.6 (14.3)	103.1 (7.7)	0.004
Verbal comprehension index	110.6 (14.0)	91.0 (15.5)	101 (10.8)	<0.001
Perceptual reasoning index	107.4 (10.3)	101.0 (19.8)	103.5 (13.1)	0.455
Processing speed index	97.2 (9.5)	96.5 (7.9)	101.9 (10.9)	0.179
Working memory index	104.9 (9.3)	96.4 (16.1)	107.1 (9.8)	0.094
BASC (range)	40–60	40–60	40–60	
Reading score (PROLEC-R)^**^				
Word reading accuracy (*n*/40)	39.6 (0.7)	36.0 (3.3)	39.8 (0.7)	<0.001
Pseudoword reading accuracy (*n*/40)	37.2 (1.7)	30.3 (5.4)	36.5 (1.8)	<0.001
Word reading speed (s)	41.1 (8.3)	82.5 (49.0)	31.9 (12.3)	<0.001
Pseudoword reading speed (s)	68.3 (15.4)	99.2 (51.3)	58.4 (19.0)	0.006
Word reading skill (accuracy/speed)×100	101.6 (25.7)	60.6 (30.9)	144.3 (45.9)	<0.001
Pseudoword reading skill (accuracy/speed)×100	57.5 (12.1)	38.3 (17.5)	70.6 (25.1)	<0.001

* Kruskal-Wallis H or Pearson's chi-square test.

** The values are mean (SD).

### Neuroanatomical measures

#### Data acquisition

The examinations were performed in a Philips Achieva 3.0-T MRI system with a 32-channel head coil (Philips Medical Systems, Best, the Netherlands). The MR scanning protocol started with an anatomical acquisition, a high-resolution structural T1-weighted 3D volume, using a spoiled gradient recalled sequence (SPGR-3D, TR/TE 7.4/3.4 ms; flip angle, 8°; matrix size, 228 × 227; field of view, 250 × 250; number of slices, 301; in plane resolution 1 × 1 × 1 mm; NSA 1). Total acquisition time: 4′58″. The structural MR scan was used for spatial corregistration and anatomical reference.

In the same scanning protocol, three consecutive sequences of BOLD functional images were acquired using an axial single shot EPI method block design (9 blocks). The following parameters were used for scanning: TR/TE 3000/30 ms; flip angle, 90°; matrix size, 96 × 96; field of view, 230 × 230 cm; slice thickness, 4 mm with no gap; number of slices, 25; number of volumes, 90; NSA 1. Total acquisition time: 4′39″.

#### Experimental design

To obtain quality data, before performing the definitive cognitive testing in the MR scanner, the participants were introduced to the cognitive tasks, its mode of presentation and functioning of the response systems in a computer system independent of the MR system. The children were shown each of the paradigms and instructed on what to do on each phase of the testing. The dummy tasks had the same structure as those performed inside the magnet, differing only in the details of the test provided to avoid repetition and learning effects.

Visual stimuli were projected onto goggles using a computer-controlled system for visual stimulus presentation (Nordic Neurolab) and all responses were recorded by means of two hand-held response boxes. All the cognitive paradigms shared the same structure, consisting of a block design: 4 blocks with 30 s of stimulus presentation, alternating with 5 control blocks of sign strings of the same duration, during which readers just watched these signs strings without no interaction.

In the first lexical decision task the participants had to read two-syllable real words or pseudowords (graphemes without actual meaning). Ten randomly presented either words or pseudowords were displayed every 3 s in each block. Once read, the participants had to decide whether the word presented corresponded to a real word or not by pressing a button on one of the response boxes they held in each hand. They had to press the right-hand button if the word corresponded to a real word and the left- hand button if the word was a pseudoword. Special emphasis was placed on the need for children to try and read pseudowords as well as they would do so for real words, despite their unfamiliar appearance.

For the second lexical/orthographic matching task two sets of two-syllable pseudowords were displayed simultaneously and the participants were asked to judge whether they were identical by pressing a button on one of the response boxes they held in each hand, the right-hand button if the pseudowords were equal and the left-hand button if they were different. Six pairs of randomly presented pseudowords were displayed every 5 s in each block. Control blocks contained six pairs of sign strings of equal morphology and length as the pseudowords presented in activation period. All the words and pseudowords presented in these first two tasks were based on the standardized reading test PROLEC-R.

In the third task, the semantic categorization task, three words were simultaneously presented, two from the same semantic category were placed at the top of the display; a third word was encased at the bottom. Children were asked to indicate whether this third word belonged to the same category as the other two or not by pressing a button on one of the response boxes they held in each hand, the right-hand button if the word belonged to the same category and the left-hand button if not. Each set of three words were presented every 5 s. Words from the same and a different semantic category were presented randomly. In the control blocks, the same structure was followed but using strings of signs of equal length instead of words.

**Figure 1:**
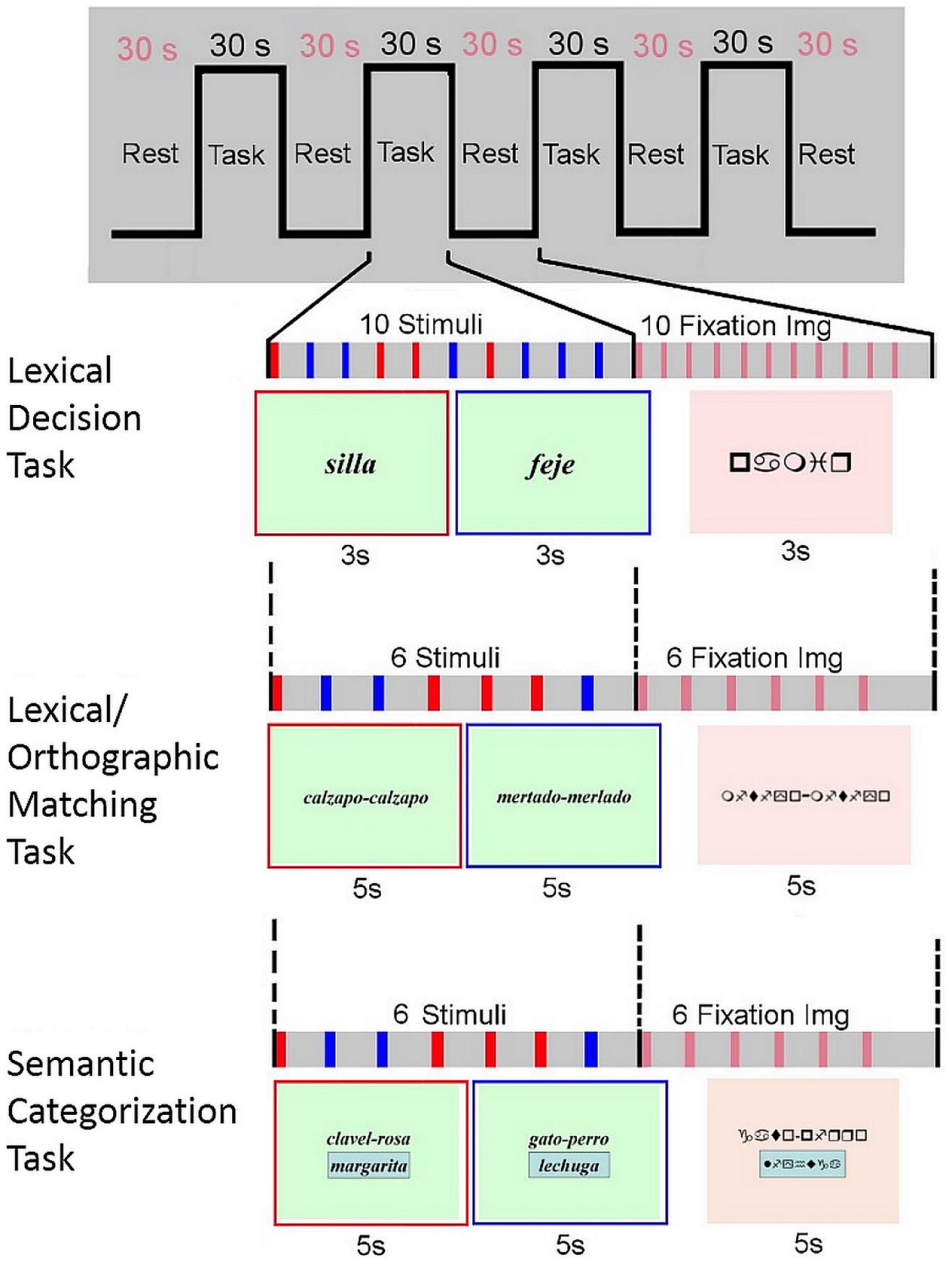
**Experimental paradigm**. For the reading blocks, in the lexical decision paradigm two kind of stimulus were visually presented, real words (e.g., *silla*, chair in Spanish), and pseudowords (e.g., *feje*, which means nothing in Spanish); in the lexical/orthographic matching paradigm two sets of two-syllable pseudowords were displayed simultaneously, either identical (e.g., *calzapo-calzapo*) or different (e.g., *mertado-merlado*); and in the semantic categorization paradigm the three words presented at the same time could be either from the same (e.g., *clavel*, *rosa*, *and margarita*; carnation, rose and daisy in Spanish) or from different (e.g., *gato*, *perro*, *and lechuga*; cat, dog and lettuce in Spanish) semantic categories.

Figure 1 outlines the design of the experimental paradigm.

#### Data processing

Descriptive statistics were used to summarize and initially inspect the distributions of demographic and study variables. Sociodemographic and neuropsychological features were compared between groups with a non-parametric test (KW test, Wilcoxon rank sum test) and Pearson's chi-square for categorical variables (SPSS v20.0; IBM Corp. Armonk, NY, USA).

#### Corregistration

The difference in size and shape characteristics between the brains of children of this age group and the most commonly used reference brain, the MNI152 (average T1 brain image constructed from 152 normal adult subjects at Montreal Neurological Institute), could induce registration biases (Hoeksma et al., 2005). To reduce this potential source of error, we corregistered the functional sequences onto a custom template specifically created for this study. First, we performed affine registration of each subject's T1 image to the MNI-space T1 standard brain template, using FLIRT (FMRIB Linear Image Registration Tool; FSL – FMRIB, Oxford, UK). Next, a custom template incorporating average size and shape characteristics of the population of participants was created using the group mean inverse transformation matrix. Finally, each subject's T1 was registered to this new custom template, and the resulting transformation matrix applied to their corresponding functional sequences (Jenkinson and Smith, 2001; de Bie et al., 2011).

All neuroanatomical landmarks are reported in Montreal Neurological Institute (MNI) reference space coordinates.

#### Individual level fMRI analysis

All fMRI analyses for each subject were performed using the general linear model implemented in FEAT (FMRI Expert Analysis Tool v5.98), part of the FSL image analysis package (FMRIB Software Library, Oxford). For all three tasks, the pre-processing of the functional MRI sequences included removal of non-brain data from both the functional and structural images of each subject (BET—Brain Extraction Tool; Smith, 2002), motion correction using a rigid-body registration MCFLIRT (motion correction FMRIB's Linear Image Registration Tool; FSL – FMRIB, Oxford, UK) (Jenkinson et al., 2002) and a highpass filter of 1/60 Hz to remove low-frequency signals. Additional spatial smoothing was applied using a Gaussian filter with 3 mm full width at half maximum.

Subject-level general lineal model analysis was carried out using FILM (FMRIB's Improved Linear Model; FSL – FMRIB, Oxford, UK), modeling each event using a double-hemodynamic response function and its temporal derivative and further applying local autocorrelation correction by prewhitening (Woolrich et al., 2001). For all tasks, stimuli not responded to or/and incorrectly answered events were not entered in the model. In addition, the word and pseudoword stimuli-related events on the lexical decision task were separately modeled to increase the precision on the experimental within-subject response.

#### Group level fMRI analysis (Inference)

The FLAME (FMRIB's Local Analysis of Mixed Effects; FSL – FMRIB, Oxford, UK) toolbox (Beckmann et al., 2003; Woolrich et al., 2004) was used to conduct the group-level analysis. All individual- and group-level fMRI results were tested using cluster-level correction with Z-score threshold >2.3, and cluster *p*-value threshold <0.05. We performed group comparisons for the three groups: first we obtained the mean group activation for each group, and then, conducted comparisons for all the pairs of groups.

We further explored functional differences between the groups in brain regions directly related to language functioning or with well-known involvement in the cognitive tasks applied. Applying the function Featquery (FSL – FMRIB, Oxford, UK) to specific regions of interest, we calculated the mean signal intensity of the corresponding ROI for every subject and compared these values between groups using SPSS (IBM Corp. Armonk, NY, USA).

The Shapiro-Wilk test was applied to evaluate the distribution of the data and Levene's test to analyse homogeneity in variance. Normality and homoscedasticity could not be assumed for all the variables studied and, to help control for Type I errors, we used a non-parametric Kruskal-Wallis (K-W) Test for comparisons between the three groups, followed by a *post hoc* Mann-Whitney (M-W) test for comparisons between pairs of groups.

To assess the relationship between functional activation and reading ability, we compared the values of cortical activation with in-scanner reading accuracy, and clinical reading accuracy and speed scores, for each condition. Correlations were analyzed using Pearson's bivariate correlation coefficient (r). No correction was applied for multiple comparisons. In some cases due to the distribution of the groups, we also split the analysis to only study correlations for specific groups.

For all the tests mentioned above a value of *p* < 0.05 was considered statistically significant.

## Results

First, the results are reported for each paradigm separately, and then certain values are compared between tasks for later discussion.

### Lexical decision

#### Group contrasts for the word reading condition

**Figure 2:**
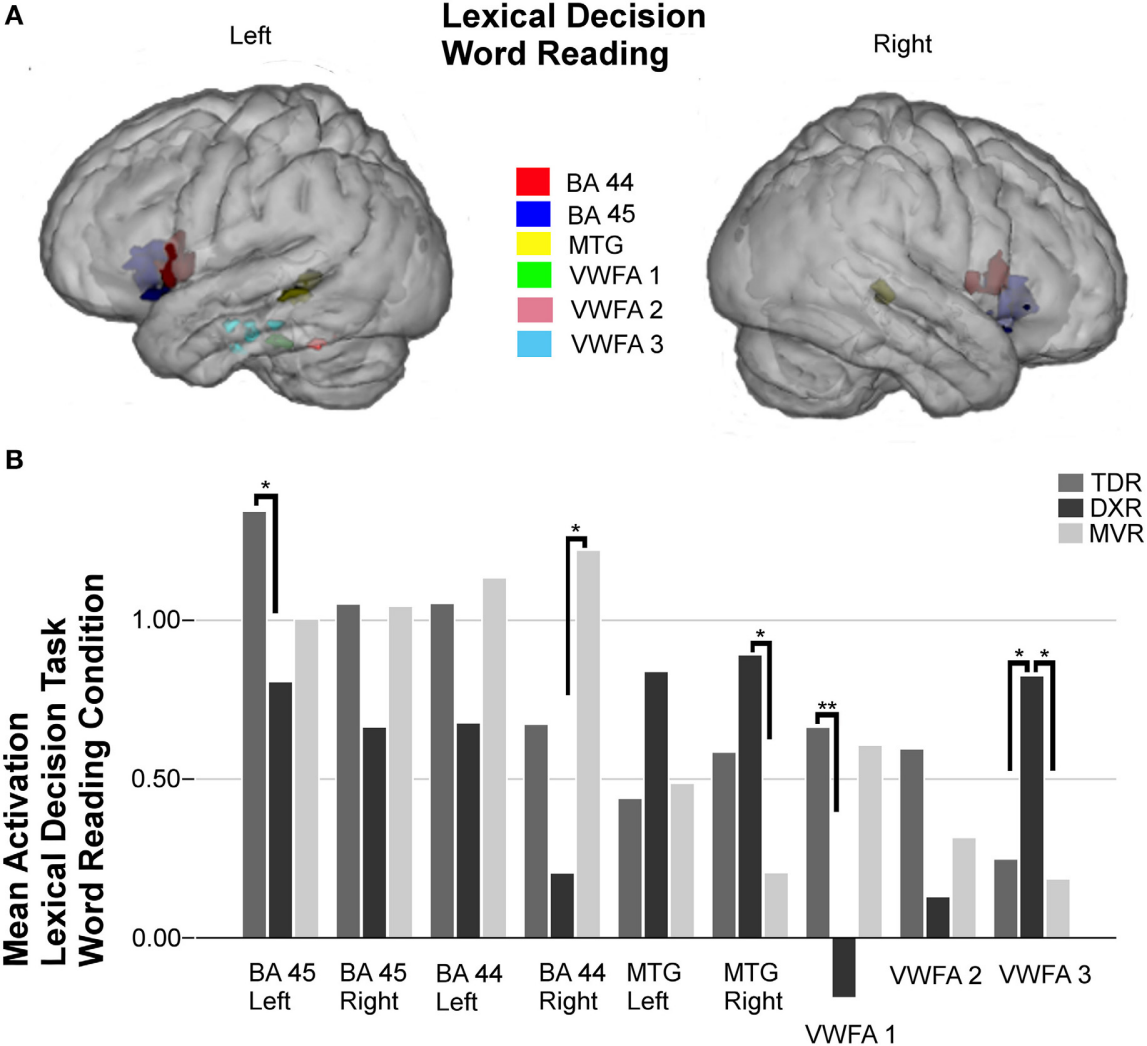
**Surface rendering of the location of the ROI evaluated in fMRI analysis (A) and group differences in mean activation for the three groups in the selected areas (B) for the word reading condition of the lexical decision task**. Left Broca's areas (BA 45: −36, 21, −1; BA 44: −53, 10, 7); right Broca's areas (BA 45: 37, 21, 1; BA 44: 53, 9, 12); left MTG (BA 21: −55, −40, 2) and right MTG (BA 21: 52, −32, 1); left VWFA 1 (BA 37: −40, −33, −25), left VWFA 2 (BA 37: −51, −48, −21), and VWFA 3 (BA 20: −45, −20, −22), ^*^P < 0.05, ^**^P < 0.01.

Nine areas were studied in the ROI analysis for this condition (Figure 2).

Dyslexic children had less activation in right Broca's areas (BA 44 and 45) and more in the left and right MTG (BA 21) than those in the other two groups, but these differences between groups were not statistically significant. Tests indicated that there were statistically significant differences in the left Broca's area (BA 45) (*p* = 0.015, M-W test).

In the left fusiform gyrus, three ROIs were identified and studied due to the activations found in previous group-level analysis; thereby, we found activation in an anterior region we call VWFA 3 (BA 20), it being more intense in dyslexics (*p* = 0.026, K-W test) than the MVRs (*p* = 0.016, M-W test) or the TDRs (*p* = 0.025, M-W test). In the other two regions, VWFA 1 (BA 37) and VWFA 2 (BA 37), located in a more posterior part of the temporal lobe, dyslexics showed marked hypoactivation compared to the other two groups, but the only significant difference was found between the TDR and DXR groups (*p* = 0.008, M-W test) in VWFA 1.

#### Correlation with scores

For the three paradigms, we performed the same comparative analysis; namely we compared the areas of cortical activation with the three variables that we expected would best reflect the children's reading ability: accuracy scores for the responses during the in-scanner tasks, and the clinical scores of accuracy and time for the execution of the pseudoword reading task in the evaluation of reading processes, since in semi-transparent languages like Spanish the reading time is as important as accuracy in reading pseudowords for the diagnosis of dyslexia (Ziegler et al., 2003).

In this word reading condition we found modest correlations (*r* < 0.4) between the above cortical activations and the in-scanner pseudoword reading accuracy or the clinical assessments scores.

#### Group contrasts for the pseudoword reading condition

**Figure 3:**
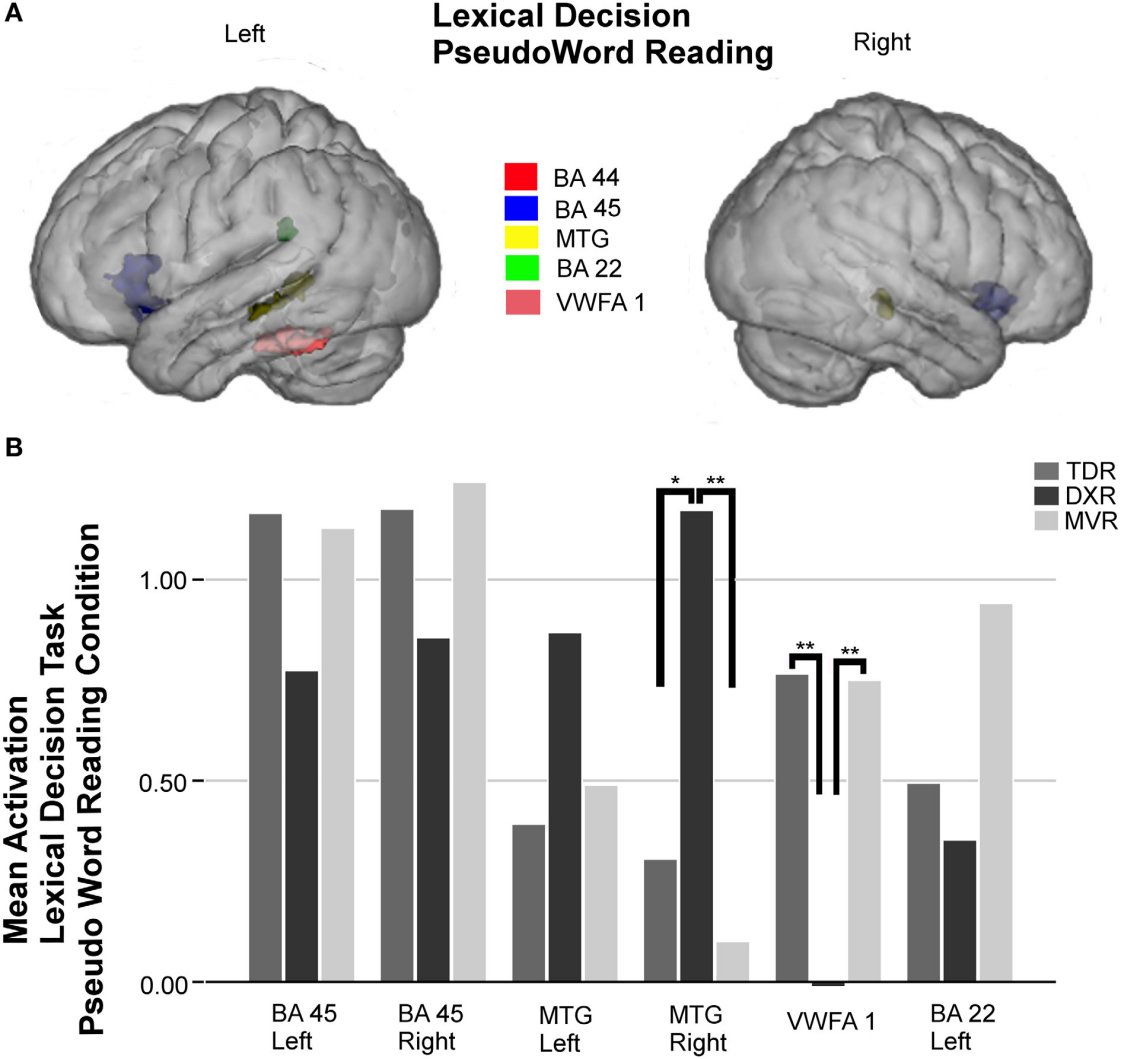
**Surface rendering of the location of the ROI evaluated in fMRI analysis (A) and group differences in mean activation for the three groups in the selected areas (B) for the pseudoword reading condition of the lexical decision task**. Parts of left and right Broca's area (left BA 45: −34, 20, −1; right BA 45: 37, 19, −4); left MTG (BA 21: −52, −39, −1) and right MTG (BA 21: 52, −32, −2); left VWFA 1 (−45, −41, −21), and left Wernicke's area (BA 22: −55, −41, 24), ^*^P < 0.05, ^**^P < 0.01.

Six areas were studied in the ROI analysis of this condition (Figure 3).

As observed in the other tasks, activation in the left BA 45 and right BA 45 was less intense in dyslexics, but no statistically significant differences were found between groups. On the other hand, there were significant differences between groups in activation in the right MTG (BA 21) (*p* = 0.004, K-W test), and comparing the DXRs with MVRs (*p* = 0.001, M-W test). The trend in the left MTG was the same as on the right side, but despite differences being notable, they were not statistically significant in this case.

In the left fusiform gyrus, there were significant differences between groups for the area we call VWFA 1 (BA 37) (*p* = 0.001, K-W test), and again, the differences were found to be significant for comparisons of DXRs with TDRs (*p* = 0.001, M-W test) and with MVRs (*p* = 0.002, M-W test) but not between MVRs and TDRs.

In the left Wernicke's area (BA 22), there was again less activation in dyslexics than the other groups but no significant differences were found.

#### Correlation with scores

We compared cortical activation in the six areas considered with the three variables mentioned before, accuracy in the in-scanner task, and accuracy and time in pseudoword reading in the clinical assessments.

In-scanner, the reading accuracy for this condition showed a significant correlation with activation in the left BA 45 (*r* = 0.600, *p* = 0.000), and also VWFA 1 (BA 37) (*r* = 0.383, *p* = 0.004).

With regard to the clinical assessments, pseudoword reading accuracy was correlated with activation in the right MTG (BA 21) (*r* = −0.386, *p* = 0.004) and VWFA 1 (*r* = 0.432, *p* = 0.001). We also found a correlation between time spent on pseudoword reading and activation of the right MTG (*r* = 0.391, *p* = 0.003) and the left Wernicke's area (BA 22) (*r* = −0.393, *p* = 0.003).

### Lexical/orthographic matching

#### Group contrasts

**Figure 4:**
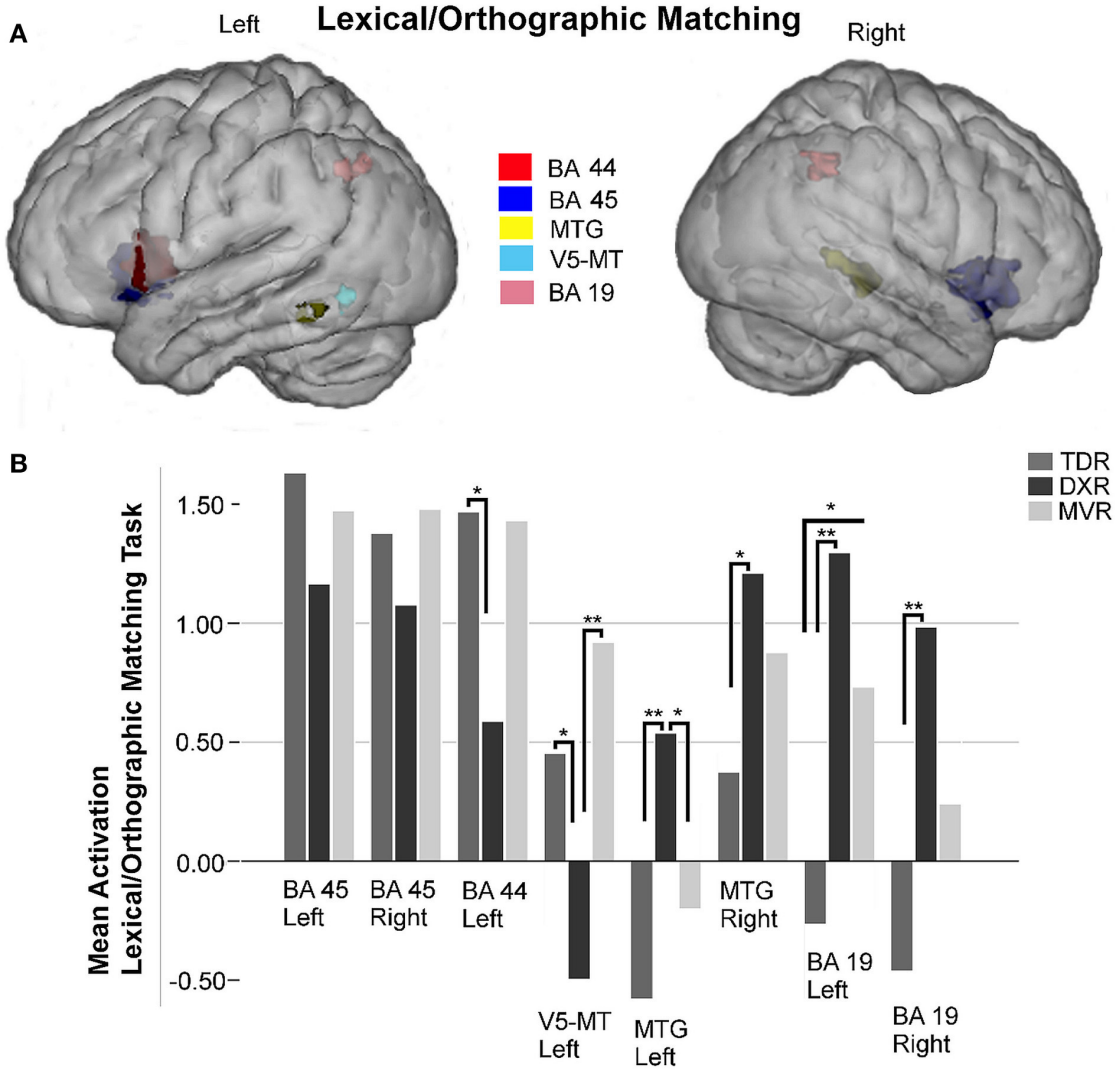
**Surface rendering of the location of the ROI evaluated in fMRI analysis (A) and group differences in mean activation for the three groups in the selected areas (B) for lexical/orthographic matching task**. Left and right Broca's areas (Left BA 45: −38, 18, −2; right BA 45: 35, 19,−2); left Broca's area (BA 44: −52, 9, 9); left V5/MT area (−44, −62, −7); left MTG (BA 21: −62, −49, −7) and right MTG (BA 21: 51, −36, 2); left and right precuneus (Left BA 19: −25, −64, 39; right BA 19: 43, −52, 41) ^*^P < 0.05, ^**^P < 0.01.

Eight areas were studied in the ROI analysis of this task (Figure 4).

In the left and right Broca's areas (BA 45) and the left BA 44 there was less activation in dyslexics than the other two groups, but the differences between groups were not statistically significant.

We did not study either Wernicke's area (BA 22) or the fusiform gyri for this paradigm because no relevant activations were observed in any of the brain-wide comparisons (group-level analysis).

Regarding the MTG, dyslexics tended to activate this area more than the other two groups, and these differences were significant for the left side (*p* = 0.009, K-W test), comparing TDRs and DXRs (*p* = 0.003, M-W test).

In the posterior part of the left MTG, the V5/MT area (BA 39) was hypoactivated in the dyslexics, with significant differences between MVRs and DXRs (*p* = 0.001, M-W test).

We found significant differences for both left and right superior parietal lobes (BA 19) (*p* = 0.002 and *p* = 0.011, both with the K-W test, respectively). For the left hemisphere, activation was significantly different comparing DXRs with TDRs (DXRs: *p* = 0.000, with the M-W test), while for the right hemisphere, significant differences were found between DXRs and TDRs (*p* = 0.003, M-W test). No differences were found in these parietal regions between DXRs and MVRs.

#### Correlation with scores

We compared eight areas with the three variables of performance.

We found a statistically significant relationship between the level of accuracy during the in-scanner task and activation in the left BA 45 (*r* = 0.505, *p* = 0.000), and the left BA 44 (*r* = 0.547, *p* = 0.000).

The accuracy in pseudoword reading revealed in the clinical assessments was significantly correlated with activation in the left BA 44 (*r* = 0.390, *p* = 0.003) and the left V5/MT (*r* = 0.349, *p* = 0.009). However, the time children spent on pseudoword reading in these assessments was only correlated with activation in left BA 44 (*r* = −0.390, *p* = 0.003).

### Semantic categorization

#### Group contrasts

**Figure 5:**
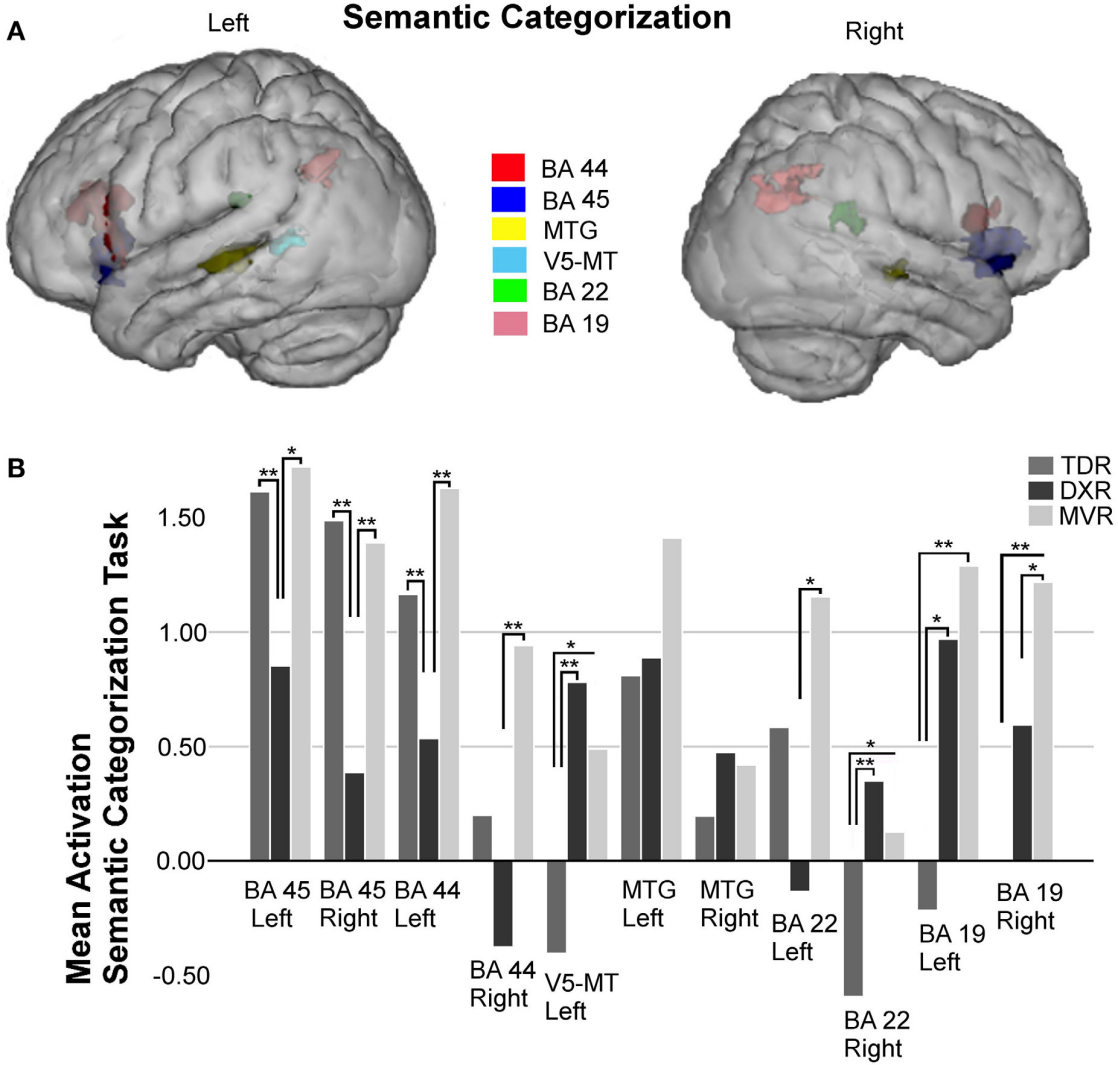
**Surface rendering of the location of the ROI evaluated in fMRI analysis (A) and group differences in mean activation for the three groups in the selected areas (B) for the semantic categorization task**. Parts of left and right Broca's area (left BA 45: −36, 19, −2; right BA 45: 40, 19, −3; left BA 44: −49, 17, 19; right BA 44: 54, 12, 14); left V5/MT area (−42, −55, 6); left MTG (BA 21: −57, −38, 2) and right MTG (BA 21: 63, −26, −1); left and right Wernicke's area (left BA 22: −61, −41, 25; right BA 22: 44, −39, 20); and left and right precuneus (left BA 19: −25, −65, 34; right BA 19: 30, −60, 35), ^*^P < 0.05, ^**^P < 0.01.

A total of 11 areas were studied in the ROI analysis of this task (Figure 5).

Significant differences between groups were found for both left and right BA 45 (*p* = 0.010 and *p* = 0.001, both with the K-W test), there being less activation in the dyslexics in both the left (TDRs vs. DXRs: *p* = 0.004, M-W test; MVRs vs. DXRs: *p* = 0.018, M-W test) and the right (TDRs vs. DXRs: *p* = 0.000, M-W test; MVRs vs. DXRs: *p* = 0.004, M-W test) hemispheres.

A similar trend was found for left and right BA 44 (left: *p* = 0.004, K-W test and right: *p* = 0.009, K-W test), the activation in dyslexics being different to that in TDRs (*p* = 0.001, M-W test) and MVRs (*p* = 0.003, M-W test) in the left BA 44, though only differences between DXRs and MVRs were significant for the right BA 44 (*p* = 0.002, M-W test).

For the left MTG, there was less activation in the TDR group than the others, while for the right MTG area there tended to be more activation in dyslexic readers than in the other two groups, but these differences were not significant.

As for the parietal lobe, between-group analysis revealed differences in the left parietal area (BA 19) (*p* = 0.007, K-W test). These differences were significant for comparisons of TDRs with MVRs (*p* = 0.002, M-W test). For the right parietal area (BA 19) (*p* = 0.003, K-W test) there were significant differences for TDRs vs. MVRs (*p* = 0.001, M-W test).

Analysis of the left Wernicke's area revealed differences (*p* = 0.033, K-W test) with far more activation in MVRs than DXRs (*p* = 0.016, M-W test), and more activation in TDR than DXR group but the differences were not significant. In the right Wernicke's area (BA 22) (*p* = 0.006, K-W test) there was more activation in dyslexics than the two other two groups, the difference being significant for TDRs (*p* = 0.002, M-W test).

For this task, we also found significant differences in the left V5/MT area, with similar levels of activation in MVR and DXR groups, both activating more than the TDR group (*p* = 0.006, K-W test) (TDRs vs. DXRs: *p* = 0.001).

#### Correlation with scores

The performance metrics for this in-scanner task revealed a statistically significant relationship between accuracy during the task and activation in both the left BA 45 and left BA 44: left BA 45 (*r* = 0.592, *p* = 0.000); right BA 45 (*r* = 0.467, *p* = 0.000) and left BA 44 (*r* = 0.574, *p* = 0.000).

From clinical scores for time measures, significant and inverse relations were observed for both the left BA 45 (*r* = −0.469, *p* = 0.000), and the left BA 44 (*r* = −0.476, *p* = 0.000).

Figures 2–5 display ROIs in the described areas for comparisons between dyslexic and non-dyslexic groups and group differences in mean activation for the three groups in the selected areas for the lexical decision task during word and pseudoword reading; the lexical/orthographic matching task and the semantic categorization task respectively.

### Differences between paradigms

In the Lexical Decision task, for the word reading condition, we found large differences between TDR and DXR groups in left VWFA 1 (*p* = 0.008, M-W test), and there was a significant correlation between activation and accuracy for reading pseudowords, from the clinical scores (*r* = 0.382, *p* = 0.004). For the pseudoword reading condition VWFA 1 was the area that reflected the best the differences between DXRs and TDRs (*p* = 0.001, M-W test). Its correlation with the accuracy for reading pseudowords, from the clinical scores, was significant (*r* = 0.432, *p* = 0.001).

In the Lexical/orthographic matching task the left Broca's area (BA 44) was the area where major differences were found between DXR and TDR groups (*p* = 0.020, M-W test), and the correlation between activation and accuracy for reading pseudowords, from the PROLEC test, was strong (*r* = 0.390, *p* = 0.003).

In the Semantic Categorization task the right Broca's area (BA 45) showed great differences between groups (TDR vs. DXR: *p* = 0.000, M-W test) and a strong correlation was observed between brain activation and the accuracy for reading pseudowords, from the clinical scores (*r* = 0.304, *p* = 0.024).

## Discussion

In our research, we have conducted a comprehensive fMRI study including three different cognitive tasks, two tasks of lexical decision and lexical/orthographic matching, read preferentially through the sublexical phonological route based on grapheme–phoneme correspondences, and one of semantic categorization, that enhances the orthographic route based on lexical units, to explore the two pathways of language in an attempt to reproduce the neural network involved in reading (Pugh et al., 1996; Saur et al., 2008; Ramus and Ahissar, 2012). Our objective was to compare the brain activation pattern while reading in children with dyslexia, children with monocular vision due to ocular motility disorders and children who were typical readers and did not have monocular vision, and assess whether they share features that would support the hypothesis that visual abnormalities are responsible for reading disorders or, conversely, dyslexia is independent of visual impairments. The combination of both lexical decision and semantic categorization tests enables a robust and detailed analysis of the reading network, particularly with the in addition of a specifically visual processing element that could highlight differences between MVR and DXR groups. Our final goal is to help to understand the underlying neurobiological dysfunction associated with dyslexia, which will contribute to children diagnosed with dyslexia receiving appropriate support and individualized evidence-based educational interventions.

First, we will summarize the results of our research and then present our hypothesis. It should first be noted that in this analysis we have only taken into account brain activation measurement from when children answered the questions posed in each task. That is, we have excluded activation when there was no response from children, to avoid bias from patterns from when they were not performing the tasks and therefore would most likely not have been activating the neural network for reading.

For the word reading condition of the lexical decision task, in line with several previous publications (Shaywitz and Shaywitz, 2003; Maisog et al., 2008; Wimmer and Schurz, 2010), our results indicate that dyslexic children tend to hypoactivate both Broca's areas and VWFA 1 and 2. Conversely, dyslexics tend to activate the posterior part of both left and right MTG and the anterior part of the VWFA, the latter in a significantly more intense way than in the other two groups.

For the pseudoword reading condition of the lexical decision task, dyslexic children were again observed to have a tendency to hypoactivate both parts of Broca's area and left Wernicke's area, and activate the left MTG more intensely; although these differences were not significant, we hypothesize that should such a pattern be confirmed, it might be a compensatory phenomenon. These differences became significant in the right MTG, dyslexic participants activating this region the most, and in VWFA 1 and 2, that dyslexics activate the least.

Comparing activation with the accuracy measures in the responses during the in-scanner task, significant correlations were seen for the left BA 45 and also for VWFA 1. Analysing our results together with the clinical scores, accuracy on pseudoword reading was positively correlated with right MTG and also left Wernicke's area activations, while time spent on pseudoword reading was negatively correlated left Wernicke's area activation and positively correlated with right MTG activation.

For the lexical/orthographic matching task, dyslexic readers again showed hypoactivation in both parts of Broca's area. In this task, as in the lexical decision paradigm described above, dyslexic readers had stronger activation in both left and right MTG, the difference being significant for the left hemisphere. It is worth noting the significant hypoactivation by the dyslexic children of the posterior part of the left MTG, the area called V5/MT. In both superior parietal lobules, BA 19, DXRs, and MVRs exhibited hyperactivation compared to TDRs, the difference being significant for the dyslexic group.

Comparing with the level of accuracy during the fMRI task, significant correlations were seen for the left Broca's areas (BA 44 and 45). Further, the accuracy on pseudoword reading was significantly positively correlated with the left BA 44 and V5/MT activation, while time spent on pseudoword reading was correlated negatively with left BA 44 activation.

Regarding the last task presented, the semantic categorization task, there are some notable findings: once again dyslexics were observed to have hypoactivation in the triangular and opercular parts of both Broca's areas (BA 44 and 45), this being statistically significant and notable. Other authors observing this pattern in children with reading disturbances have attributed it to a deficit in semantic integration (Booth et al., 2007). They hypoactivate the left Wernicke's area, activating it significantly less than MVRs, and seem to balance this with a greater activation of the contralateral, right, Wernicke's area. In this paradigm both MVRs and DXRs hyperactivate the left V5/MT and left and right parietal areas (BA 19) compared to TDRs.

Another finding we would like to highlight is the difference observed in the activation of the two MTG. In the left hemisphere, dyslexics had a similar level of cortical activation to controls, and less activation than MVRs; while in the right hemisphere, dyslexics were seen to have enhanced activation, greater than in controls but quite similar to that in MVRs.

As for performance on the in-scanner task, results revealed statistically significant relationships between scores and activations across left BA 45 and left BA 44. Considering clinical scores, the time spent on pseudoword reading was inversely related to both left BA 44 and 45.

Outlining our results, overall, for the three paradigms used, the pattern of activation while reading in MVRs seems differ from that in DXRs but be similar to that in TDRs. In accordance with other studies (Paulesu et al., 2001; Georgiewa et al., 2002; Shaywitz and Shaywitz, 2003; Pernet et al., 2009), our findings suggest that in relation to the two paradigms designed to explore the phonological route, dyslexic children tend to hypoactivate some of the more areas in the left hemisphere engaged by the phonological route, especially the posterior part of the VWFA, key in prelexical processing, the left Wernicke's area (for the pseudoword reading condition), and both Broca's areas.

A hypothetical explanation for the pattern observed is that DXRs compensate for this deficit related to the phonological route by activating an anterior part of the VWFA and the posterior part of both MTG, all of them related to the orthographic route, more strongly than other children. That is, in line with other authors (Hoeft et al., 2011), we believe that dyslexics may compensate for an impairment in the phonological route through the orthographic routes of both hemispheres.

The hypoactivation of both inferior frontal gyri we observed in the DXR group is consistent with previous studies in children (Georgiewa et al., 1999; Shaywitz et al., 2002), as opposed to the hyperactivation of these inferior frontal gyri observed in studies performed in adults with dyslexia (Shaywitz et al., 1998; Brunswick et al., 1999). This observation suggests the hypothesis that some compensatory mechanisms develop over time, an issue of great significant relevance regarding the importance of an early and specific treatment for these children.

The VWFA is considered by many authors to be the main specific prelexical node, in charge of processing words and word-like stimuli. As such, it would play the role of segmenting, classifying, and relaying visual word information to other regions for further analysis (Jobard et al., 2003; Yeatman et al., 2013). It would be linked with either the dorsal phonological or ventral orthographical routes depending on which part of the gyrus is being activated. Some studies have demonstrated more activation in its posterior part during pseudoword reading (the phonological route) (Dietz et al., 2005) and in its anterior part during word reading (the orthographic route) (Brunswick et al., 1999; Nakamura et al., 2005). We observed activation of the left fusiform gyrus predominantly for reading words and pseudowords and not in the other tasks, in line with the theory of the specialization of this area as a prelexical node. Notably, however, we also found a differential activation pattern between groups in three regions within this area, with greater activation in DXRs in an anterior region (VWFA 3) which would correspond to what other authors have referred to as LIMA (Lateral Inferior-temporal Multimodal Area), more activated in the that an interface area linked to the phonological, orthographic and semantic information; and a posterior region mostly activated in the non-dyslexic group (VWFA 1), which corresponds to the actual VWFA, supporting the theory of the subspecialization of this left fusiform gyrus (Cohen et al., 2004; Devlin et al., 2006; Danelli et al., 2013).

It has been hypothesized that the VWFA is indirectly connected to the phonological system by short U-shaped fibers, which project to the Wernicke's area (Catani and Mesulam, 2008), and connected by the IFOF (Inferior Fronto-Occipital fasciculus) to the semantic regions (Epelbaum et al., 2008; Martino et al., 2010), enabling access to the meaning and properties of words read (Jobard et al., 2003; Vandermosten et al., 2012). Nevertheless, these two functionally-segregated networks naturally interact closely, to obtain a high proficiency in reading fluently and comprehending a written text (McCandliss et al., 2003; Heim et al., 2005; Saur et al., 2008).

The dorsal phonological route is associated with word access through grapheme-to-phoneme mapping, preferential for reading pseudowords, kana, or uncommon words, or for phonological judgments. The ventral orthographic route is used to read commonly occurring or orthographically irregular words, and enables access to the meaning and properties of words read (Jobard et al., 2003; Ortiz-Siordia et al., 2008; Vandermosten et al., 2012). Considering our results, it is plausible that children with dyslexia read pseudowords as if they were irregular words and, if so, they would see them like a figure, memorizing without decoding them, and would also make phonological judgments through the orthographic route.

If we focus on the paradigm linked to the orthographic route, as has been suggested by previous authors (Shaywitz and Shaywitz, 2003; Pugh et al., 2010), dyslexic readers repeatedly hypoactivated the left Wernicke's area (related to phonological decoding) and, on both sides, the triangular part of Broca's area (related to working and semantic memory), and interestingly they seem to compensate by activating the Wernicke's area of the contralateral hemisphere. Nevertheless, it should be emphasized that in contrast to other studies that found hypoactivation in both MTG during a semantic task (Shaywitz et al., 2002), in our research the activation of the MTG is similar to that in the other two groups. A possible explanation for this finding is that in this paradigm all three groups use the orthographic route preferentially. Therefore, unlike other in other studies (Landi et al., 2010), we have obtained different brain pattern dysfunctions depending on whether the task performed was phonological or semantic.

As for the visual magnocellular pathway, our results are in line with previous studies showing that this area is not critical for reading single words or pseudoword (Danelli et al., 2013), since we have only obtained activation of this area in lexical/orthographic matching and semantic categorization tasks, but are contrast with other studies that have found hypoactivation in some areas along this pathway (Simos et al., 2000; Hoeft et al., 2007; Heim et al., 2010; Reilhac et al., 2013). In the lexical/orthographic matching task dyslexic readers significantly hypoactivated the posterior part of the left MTG, the area called V5/MT that is related to motion processing. However, in both superior parietal lobules, BA 19, which belong to the same pathway as V5/MT area, there was hyperactivation in the DXR and MVR groups relative to TDR, as has been already observed in previous studies (Backes et al., 2002; Menghini et al., 2006).

For reading words in the semantic categorization task, both DXRs and MVRs hyperactivated the left V5/MT area compared to TDRs. It could be concluded that while reading words the left V5/MT and both left and right BA 19, all belonging to the visual magnocellular pathway, are the only regions in which dyslexic readers and readers with monocular vision secondary to ocular motility disorders have the same cortical activation pattern, but they differ markedly in the pattern of activation of the neural network for reading. Both groups hyperactivate the visual magnocellular pathway, which we hypothesize to be a form of compensation, in children with functional monocular vision secondary to visual sensory deficits, and in case of children with dyslexia secondary to an impairment in the neural network for reading. Our results would support the hypothesis proposed in recent articles published on this topic (De Luca et al., 2002; Roach and Hogben, 2004; Olulade et al., 2013) that visual magnocellular dysfunction is not causal to dyslexia but rather the consequence of a hampered reading.

Taking into account the correlations we found with the clinical scores, such as accuracy in pseudoword reading being correlated positively with VWFA 1 and 2 and right MTG activation, and negatively with VWFA 3, for the lexical decision task; and the positive correlation of accuracy in pseudoword reading with left Broca's area activation in the lexical/orthographic matching task, among others, our data are consistent with evidence in the literature until now (Shaywitz et al., 1992, 2003), in the sense that there is no clear boundary between dyslexics and typical readers. That is, reading skills lie on a continuum with no clear distinction between typically developing readers and readers with dyslexia, with dyslexics representing the lower tail of a normal distribution of reading ability.

A final question we asked at the start of this research was whether we could define an optimal paradigm and a corresponding brain area with strongly significant discrimination for the study of dyslexia. A definitive answer is beyond the scope of this study, as it would need more exhaustive research in a larger population, but based on the strongest correlations we found between clinical scores and brain activation areas among these three paradigms, if we wanted to analyse a population of dyslexics we would expect to obtain the best results using the paradigms of semantic categorization, examining the right Broca's area (BA 45), and lexical decision for the pseudoword reading condition, focusing our attention on the VWFA.

Our study has several limitations. First of all, recognizing that dyslexia is a complex condition that is not associated with a single phenotype and therefore that it would be simplistic to assume it could be characterized by a single neurological abnormality, we have focused only on ocular motility disorders as a hypothetical cause of dyslexia. Other potential causes in the area of vision, such as the visual attention span deficit were beyond our scope of this study and have not been taken into account in developing our hypotheses or drawing our conclusions.

Further, our sample is composed of a relatively small number of children, and we have not taken into account the possible subtypes of dyslexia. Given this, more studies with larger samples of children and different experiments comparing different profiles of dyslexic children are needed to obtain a higher level of evidence. Moreover, we have focused our research on the differences observed between the three groups of children in cortical activation patterns from the reading network. Other cortical activations outside this network, while possibly interesting, have not been taken into consideration and should also be explored in future research.

In conclusion, according to our results, Spanish-speaking children with dyslexia do not share the same brain network for reading as those with impaired binocular vision due to ocular motility disorders except in the visual magnocellular pathway. In particular, dyslexic readers appear to have a more impaired phonological route and we hypothesize that they may try to compensate for this by activating the reading network of the contralateral hemisphere and both orthographic routes.

Ocular motility disorders would not be a causal factor for dyslexia. In particular, visual magnocellular dysfunction would not be causal of dyslexia but rather the consequence of a hampered reading. Hence, even though the treatment must be always multidisciplinary, it should be based on improving phonological awareness and language development.

## Funding

This research was partially supported by the Spanish Government (Carlos III Health Institute, FIS Project PI08/01684) (URL: www.isciii.es) and the publication fees by the Basque Government Department of Education (eVIDA Certified Group IT579-13) (URL: www.hezkuntza.ejgv.euskadi.net).

### Conflict of interest statement

The authors declare that the research was conducted in the absence of any commercial or financial relationships that could be construed as a potential conflict of interest.

## Footnotes

^1^http://www.ncld.org

## Abbreviations

BA: Brodmann area
DXR: dyslexic reader
fMRI: functional MRI
IQ: intelligence quotient
K-W: Kruskal-Wallis
MTG: middle temporal gyrus
MVR: reader with monocular vision secondary to ocular motility disorders
M-W: Mann-Whitney
ROI: regions of interest
TDR: typical reader
V5/MT area: V5/medium temporal area
VWFA: visual word form area.
