# Supplementary material for: Reading networks in children with dyslexia compared to children with ocular motility disturbances revealed by fMRI
Source: Front Hum Neurosci. 2014 Nov 19;8:936. doi: 10.3389/fnhum.2014.00936 (PMC4237045; doi:10.3389/fnhum.2014.00936)
Supplement: Supplementary file 1 [file Presentation1.PDF]

Supplementary Material

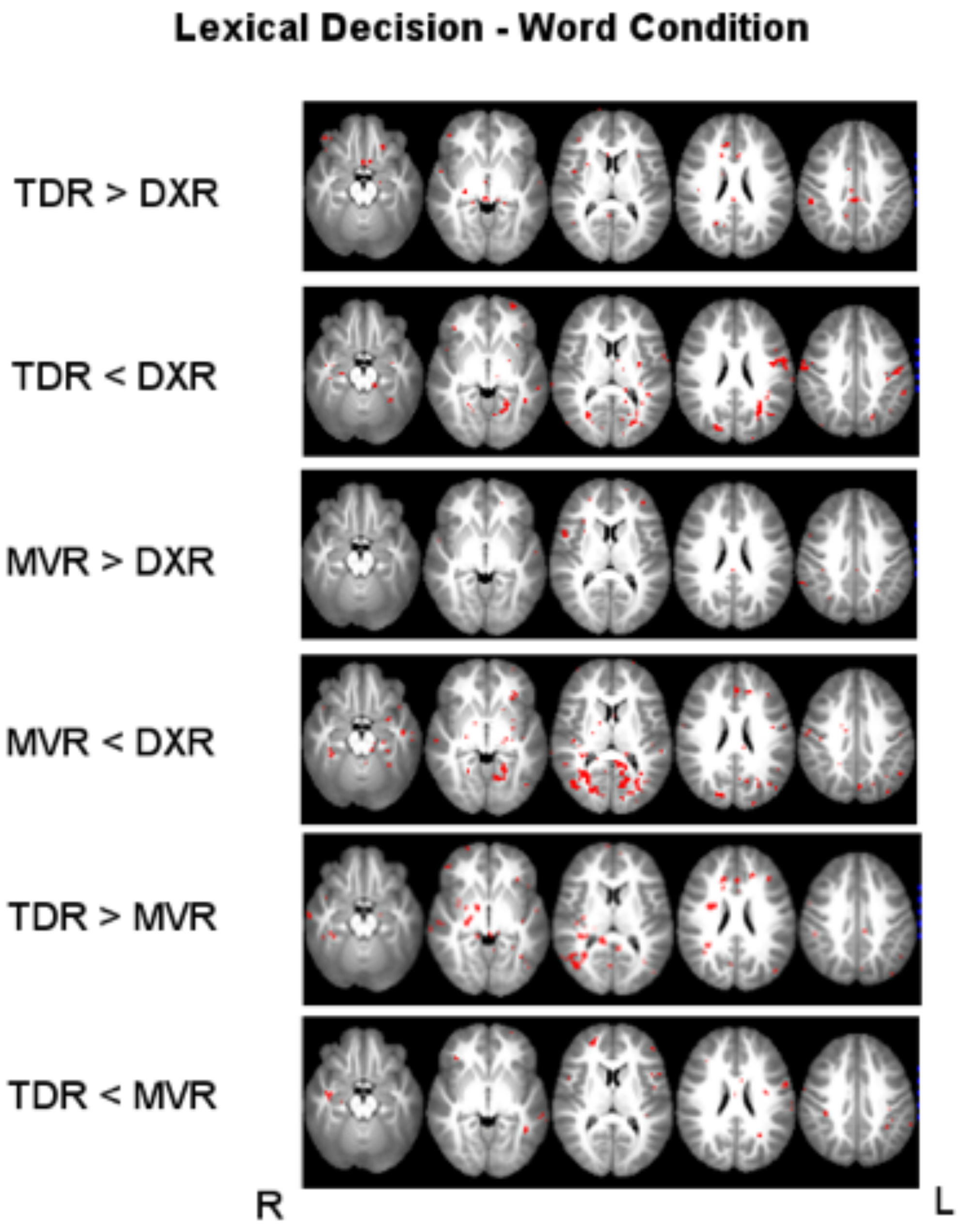

Group differences in mean activation among the three groups for the lexical decision task during word reading condition. Images are presented at an uncorrected threshold of $Z > 2.3$. Each line displays one multi-slice image from right to left slices at MNI z coordinates: -28, -4, 10, 24, 38.

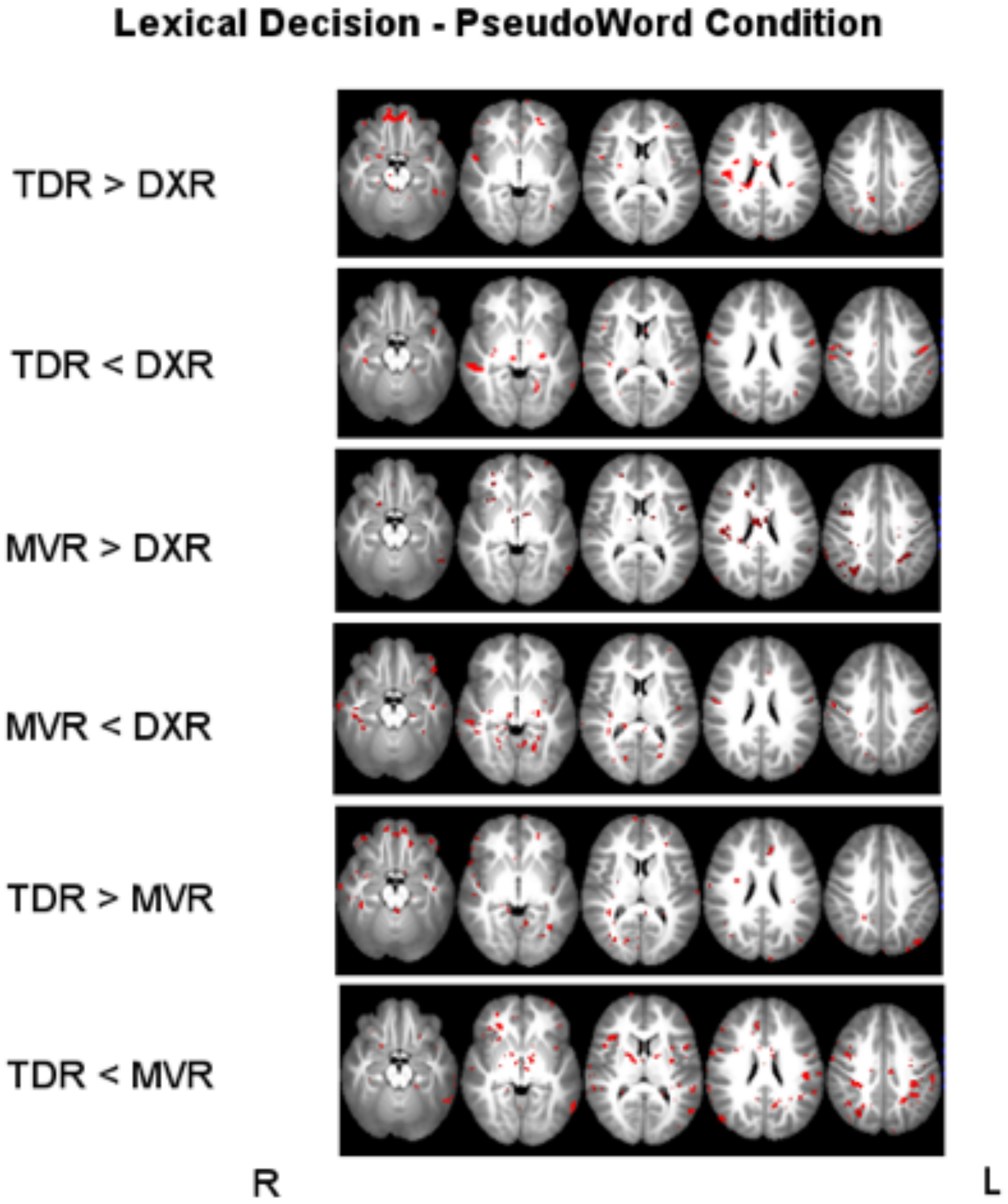

Group differences in mean activation among the three groups for the lexical decision task during pseudoword reading condition. Images are presented at an uncorrected threshold of Z > 2.3. Each line displays one multi-slice image from right to left slices at MNI z coordinates: -28, -4, 10, 24, 38.

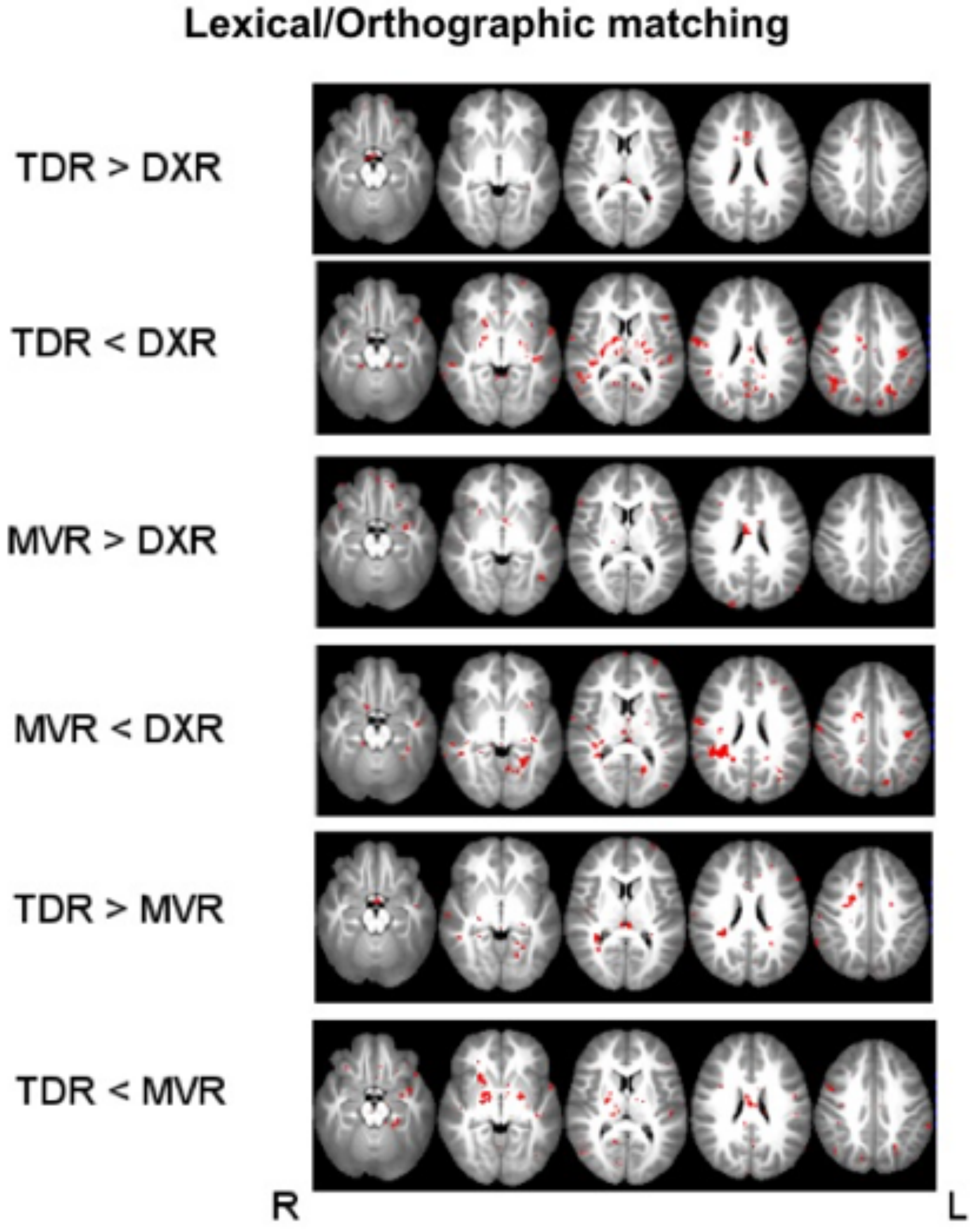

Group differences in mean activation among the three groups for the lexical/orthographic matching task. Images are presented at an uncorrected threshold of Z > 2.3. Each line displays one multi-slice image from right to left slices at MNI z coordinates: -28, -4, 10, 24, 38.

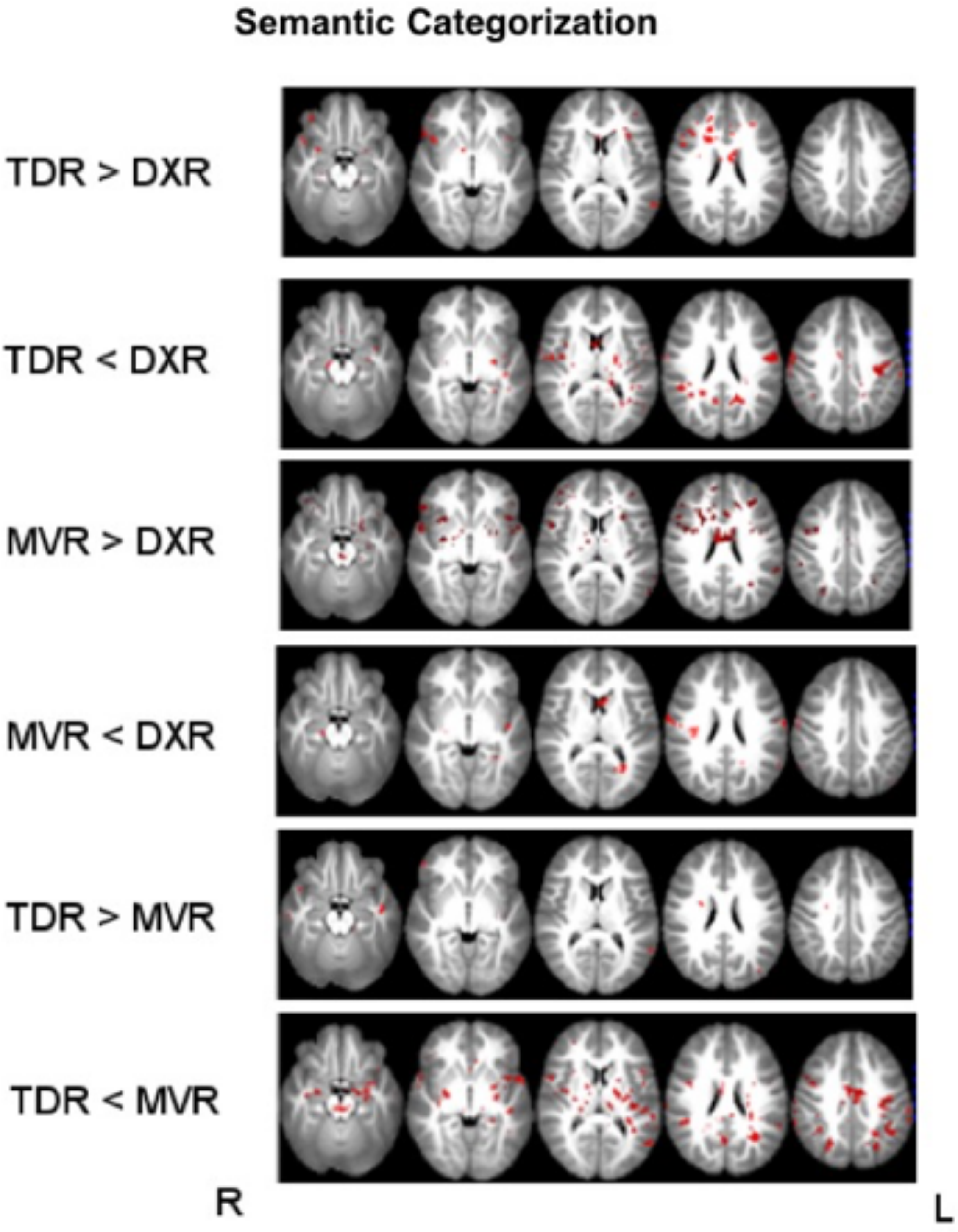

Group differences in mean activation among the three groups for the semantic categorization task. Images are presented at an uncorrected threshold of Z > 2.3. Each line displays one multi-slice image from right to left slices at MNI z coordinates: -28, -4, 10, 24, 38.

Table I. Lexical Decision Task. Word Reading Condition. fMRI Group contrasts.

Contrast	Area	Peak Z - Value	MNI Coordinates
TDR > DXR	Left Fusiform gyrus	2.96	-44, -32, -22
	Right Cuneus	3.72	52, -66, 2
	Left Inf Temporal gyrus	2.86	-41, -64, -8
TDR < DXR	Left V5-MT area	3.17	-45, -50, -4
	Left Hippocampus	4.22	-22 -54, -4
	Left Parietal lobe	4.06	-28, -56, 26
	Right Parietal lobe	3.35	30, -68, 10
MVR > DXR	Left Fusiform gyrus	2.64	-37, -32, -26
	Right PreFrontal area	3.52	16 58, 4
MVR < DXR	Left Inf Temporal gyrus	2.34	-59 -22, -22
	Right Parietal lobe	4.28	-22, -76, 12
	Left Parietal lobe	4.24	42, -74, 18
TDR > MVR	Right Fusiform gyrus	2.88	40, -34, -20
	Left Fusiform gyrus	2.87	-43, -32, -20
	Right Parietal lobe	3.22	36 -74 20
	Right MTG	3.21	52, -22, -6
	Left Parietal lobe	3.72	-36, -42, 47
TDR < MVR	Left Precentral gyrus	3.06	-59, 4, 20
	Left MTG	3.86	-67, -40, -6

Z>2.3 and Uncorrected Threshold

Table II. Lexical Decision Task. Pseudoword Reading Condition. fMRI Group contrasts.

Contrast	Area	Peak Z- Value	MNI Coordinates
TDR > DXR	Left Fusiform gyrus	4.10	-53, -42, -22
	Right Broca area	3.58	50, 2, 0
TDR < DXR	Right MTG	4.18	46, -34, -2
	Right PreCentral gyrus	3.43	63, 2, 24
	Left PreCentral gyrus	3.86	-53, -12, 40
MVR > DXR	Left Parietal lobe	3.95	-36, -34, 47
	Left Fusiform gyrus	3.58	-53, -44, -24
	Right PreFrontal area	3.40	30, 38, -6
	Left Wernicke area	2.82	-55, -42, 28
MVR < DXR	Right MTG	4.50	56, -22 -8
	Right Fusiform gyrus	3.84	38, -24, -20
	Left Cuneus	3.66	-39, -80, 18
TDR > MVR	Right Fusiform gyrus	3.30	58, -24, -16
	Left Fusiform gyrus	3.10	-41, -12, -26
	Right Sup Temporal gyrus	3.22	48, -58, -0
TDR < MVR	Right Parietal lobe	3.05	34, -34 40
	Left Parietal lobe	3.88	-32 -34, 49
	Left Occipital lobe	2.94	-53, -64, 6
	Right Broca area	3.38	34, 30, -14

Z>2.3 and Uncorrected Threshold

Table III. Lexical/Orthographic Matching Task. fMRI Group contrasts.

Contrast	Area	Peak Z - Value	MNI Coordinates
TDR > DXR	Left Broca, BA 44	4.15	-49, 6, 6
	Left V5-MT area	3.90	-41, -62, -10
TDR < DXR	Left MTG	2.77	-63, -34, 4
	Right MTG	4.03	54, -46, 12
	Right Parietal lobe	3.43	42, -52, 38
	Left Parietal lobe	3.96	-24, -60, 38
MVR > DXR	Left V5-MT area	3.16	-45, -60, -6
MVR < DXR	Left MTG	3.02	-67 -42, 6
	Right MTG	3.15	58, -32, -4
TDR > MVR	Left Frontal lobe	3.42	-22, 52, -14
TDR < MVR	Left Parietal lobe	2.67	-24, -64, 38
	Right Precentral gyrus	3.15	46, 2, 43

Z>2.3 and Uncorrected Threshold

Table IV. Semantic Categorization Task. fMRI Group contrasts.

Contrast	Area	Peak Z - Value	MNI Coordinates
TDR > DXR	Left Broca area	3.28	-39, 18, 4
	Right Broca area	3.35	54, 24, -4
	Left Occipital lobe	3.03	-61, -56, 10
TDR < DXR	Left V5-MT area	4.45	-47, -54, 6
	Left Frontal lobe	3.60	-28, 32, -12
	Right Wernicke area	4.41	42, -36, 16
MVR > DXR	Right Broca area	4.27	56, 4, -2
	Left Broca area	3.23	-41, 18, -2
MVR < DXR	Right PostCentral gyrus	4.54	60, -8, 22
TDR > MVR	Left Sup Temporal gyrus	3.10	-47, -10, -18
TDR < MVR	Left V5-MT area	3.64	-51, -58, -0
	Left Parietal lobe	4.66	-32, -58, 24
	Right Parietal lobe	3.74	26, -60, 32
	Left Broca area	4.41	-45, 8, -8

Z>2.3 and Uncorrected Threshold
